# Heart of Endosymbioses: Transcriptomics Reveals a Conserved Genetic Program among Arbuscular Mycorrhizal, Actinorhizal and Legume-Rhizobial Symbioses

**DOI:** 10.1371/journal.pone.0044742

**Published:** 2012-09-06

**Authors:** Alexandre Tromas, Boris Parizot, Nathalie Diagne, Antony Champion, Valérie Hocher, Maïmouna Cissoko, Amandine Crabos, Hermann Prodjinoto, Benoit Lahouze, Didier Bogusz, Laurent Laplaze, Sergio Svistoonoff

**Affiliations:** 1 Laboratoire Commun de Microbiologie IRD/ISRA/UCAD, Centre de Recherche de Bel Air, Dakar, Senegal; 2 Institut de Recherche pour le Développement (IRD), UMR DIADE, Equipe Rhizogenèse, Montpellier, France; 3 Department of Plant Systems Biology, VIB, Ghent, Belgium; 4 Department of Plant Biotechnology and Genetics, Ghent University, Ghent, Belgium; Friedrich-Alexander-University Erlangen-Nurenberg, Germany

## Abstract

To improve their nutrition, most plants associate with soil microorganisms, particularly fungi, to form mycorrhizae. A few lineages, including actinorhizal plants and legumes are also able to interact with nitrogen-fixing bacteria hosted intracellularly inside root nodules. Fossil and molecular data suggest that the molecular mechanisms involved in these root nodule symbioses (RNS) have been partially recycled from more ancient and widespread arbuscular mycorrhizal (AM) symbiosis. We used a comparative transcriptomics approach to identify genes involved in establishing these 3 endosymbioses and their functioning. We analysed global changes in gene expression in AM in the actinorhizal tree *C. glauca*. A comparison with genes induced in AM in *Medicago truncatula* and *Oryza sativa* revealed a common set of genes induced in AM. A comparison with genes induced in nitrogen-fixing nodules of *C. glauca* and *M. truncatula* also made it possible to define a common set of genes induced in these three endosymbioses. The existence of this core set of genes is in accordance with the proposed recycling of ancient AM genes for new functions related to nodulation in legumes and actinorhizal plants.

## Introduction

Mutualistic interactions between plants and microorganisms are an essential and widespread adaptive response whose origin can be traced back to land colonisation by plants: fossil evidence demonstrates that ∼450 million years ago primitive plants were already associated with fungi to form arbuscular mycorrhizal (AM) symbioses [1]. Today, more than 80% of terrestrial plants form AM in association with Glomeromycota fungi. AM fungi colonise the root cortex and differentiate intracellular structures inside cortical cells – arbuscules or coiled hyphae – which play a crucial role in nutrient exchange. AM significantly improve plant mineral nutrition, increasing growth and tolerance to environmental stresses including pathogens [2].

More recently, ∼60 MY ago, certain plants evolved the ability to form endosymbiotic associations with nitrogen-fixing bacteria to improve their nitrogen acquisition. The most intricate of these symbioses leads to the formation of a new organ, the root nodule, where bacteria hosted in a favourable environment inside plant cells are able to fix enough atmospheric nitrogen to sustain plant growth without any other nitrogen source. The ability to form root nodule symbioses (RNS) evolved only in fabids and gave rise to two main types of symbioses: (1) rhizobial RNS involve gram negative proteobacteria collectively called rhizobia that associate with plants from the *Fabaceae* superfamily and a few species from the genus *Parasponia* (*Cannabaceae*), (2) actinorhizal symbioses combine fabids distributed into 8 families, collectively called actinorhizal plants, and the gram positive actinomycete *Frankia* [3]–[5]. Nodulation emerged several times independently within the Fabidae suggesting that the common ancestor of this clade acquired a still-unknown predisposition towards RNS [4]. Most genes involved in nodulation are similar to genes involved in other processes, suggesting that RNS evolved by recycling a variety of pre-existing genetic mechanisms. Genes controlling the development of rhizobial infection threads are probably derived from genes controlling pollen tube growth [6]. Many genetic mechanisms making it possible to accommodate symbiotic bacteria originate in more ancestral AM symbiosis [4], [7], [8]: the symbiotic signals emitted by rhizobia and AM fungi are chemically related [9]. In addition, part of the signalling pathway responsible for signal transduction in host plants in response to recognition of the microbial partner is shared between AM, rhizobial and actinorhizal symbioses [8], [10]–[13].

We used comparative transcriptomics to identify genes induced during AM and nodulation (actinorhizal or rhizobial) in several plants, including legumes [14], [15], rice [16] and the actinorhizal tree *Casuarina glauca* [17]. As no data on AM in actinorhizal plants were available, we characterised the establishment of AM between the actinorhizal tree *Casuarina glauca* and *Glomus intraradices* and analysed its transcriptome profile. By comparing genes induced in AM in *Medicago truncatula,* rice and *C. glauca* we identified a group of genes induced in AM in these three distant species and a group of genes induced during AM, rhizobial and actinorhizal nodulation. Those genes were clustered in functional groups that may play crucial roles in the establishment and the functioning of the three endosymbioses and how they work.

## Results and Discussion

### Establishment of AM symbiosis between *C.glauca* and *G. intraradices*

**Figure 1:**
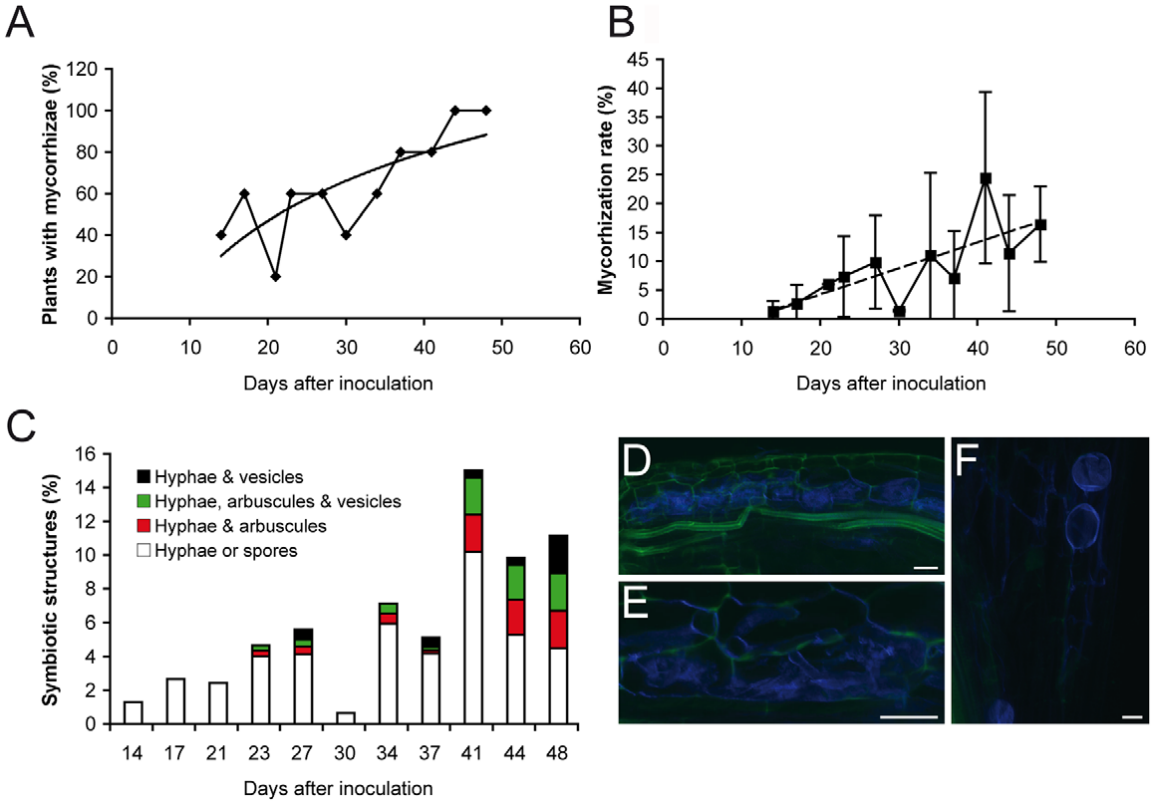
Analysis of AM establishment in C. glauca. (A) Percentage of plants showing internal AM structures; (B) Average mycorrhization rate in plants showing internal AM structures (bars: standard deviation); (C–E) Analysis of intraradical structures in roots of *C. glauca* roots after inoculation with G. intraradices: (C): quantitative analysis (D–F) CLSM images acquired on roots 45 days after inoculation showing extensive fungal colonisation, the presence of arbuscules (D), coiled hyphae (E) and vesicles (F). Bar = 20 mm.

First we characterised the colonisation kinetics of *C. glauca* by the AM fungus *G. intraradices*. Three-week old plants were transferred to pots containing soil inoculated with *G. intraradices*. Five plants were analysed for their mycorrhizal status every 3 days from two weeks after inoculation to 48 days after inoculation (dai). We observed a regular increase in the percentage of plants showing intraradical fungal structures over time; all plants were colonised from 44 dai (Figure 1A–B). The type of fungal structures observed on the plant root varied over time. Up to 21 dai only intraradical hyphae were observed. At 23 dai, coiled hyphae, arbuscules and vesicles appeared (Figure 1C-E). From these observations, plants 45 dai were selected to characterise the *C. glauca* transcriptome response to AM symbiosis.

### Gene expression in *C. glauca* AM and comparison with other AM symbioses

In order to identify the *C. glauca* genes regulated by AM symbiosis, a 15 K *C. glauca* genechip [17] was hybridised with cDNA from control (non inoculated) and roots inoculated with *G. intraradices*. 124 genes were down-regulated and 430 up-regulated in *C. glauca* AM roots (FC≥2, p-value≤0.01). Microarray data were confirmed by Q-PCR on genes showing various expression levels (Table S1). We were particularly interested in identifying genes involved in the intracellular accommodation of symbionts. While down-regulation of some gene might be important for intracellular accommodation of symbionts (for instance defence-related genes), we focused our analysis on genes that were induced. Of these, 324 appeared to be from *C. glauca* and 106 from *G. intraradices* (Tables S2 and S3). Homologues of known specific AM marker genes *PT4* (Phosphate transporter 4), *BCP1* (Blue Copper Protein 1), or *SCP1* (Serine CarboxyPeptidase 1) [15], [18] were induced in our dataset, thus validating the experiment (Table S2). CGCL918Contig1, a presumed homologue of the aquaporin NIP1 (Nodulin 26-like intrinsic protein 1) specifically expressed at a low level in the arbuscule-containing cells [18] was also induced in our data. This might suggest that our experimental set made it possible to detect genes with low levels of expression.

**Figure 2:**
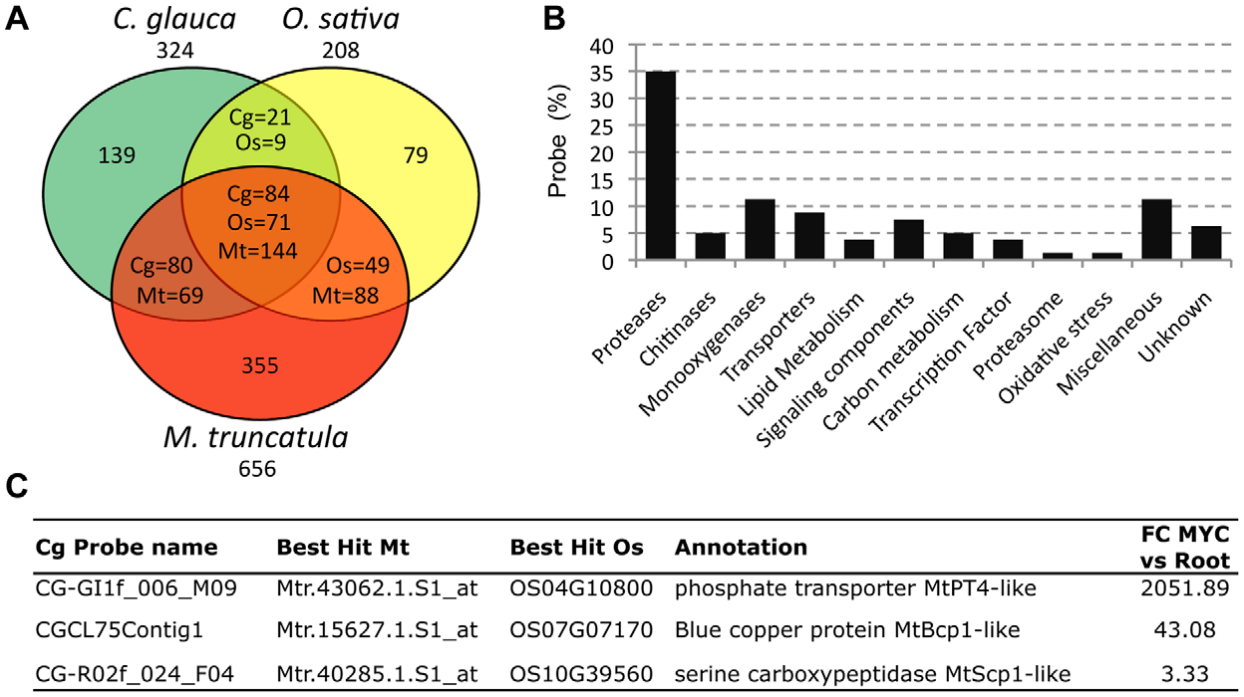
Transcriptional regulations in *M. trucatula*, *O. sativa* and *C. glauca* AM. (A) Number of genes up-regulated in AM in these different species; (B) Functional distribution of the 84 AM-induced genes in *C. glauca* and conserved in *M. truncatula* and *O. sativa*; (C) Induction of AM markers in *C. glauca* 48 days after inoculation by *G. intraradices.*

We then compared genes up-regulated in AM in *C. glauca*, the model legume *M. truncatula* [15] and the monocot *O. sativa* [16]. This analysis revealed 84 *C. glauca* genes up-regulated in AM similar to *M. truncatula* and *O. sativa* AM-induced genes (Figure 2A–C, Table S4). These may represent some core functions needed for AM symbiosis.

The cluster most represented corresponded to proteases (27 *C. glauca* unigenes), in accordance with the important role played by protein turnover in AM [16], [19]–[21]. Among the conserved proteases, we found subtilisin proteases of the S08A family. None of these correspond to *Cg12* the *C. glauca* subtilase that is specifically expressed during plant cell infection by *Frankia* [22], [23]. In *Lotus japonicus*, two members of this family, LjSbtM1 and LjSbtM3, are found in the peri-fungal space and are involved in AM development [24]. Proteases may be responsible for selective processing of substrates present in the peri-fungal space, generating peptides recognised by leucine-rich repeat receptors involved in the AM interaction such as PaNFP or SYMRK. Interestingly, a gene encoding a putative receptor with LRR repeats (CG-R02f_036_O05) was induced in AM in all three plants. Proteases may also be involved in the cell wall loosening and cell remodelling associated with mycorrhizal infection [24], or in arbuscule senescence [25]. Ten *C. glauca* sequences corresponding to carboxypeptidases belonging to the papain C1A family [26] may belong to this category as this family contains senescence-associated proteins such as AtSAG12 and MtCP1-6 [25].

Seven genes encoding putative members of the cytochrome P450 family were among the conserved genes. Most of them belong to the CYP71 family that is usually associated with triterpenoid biosynthesis. Triterpenoids play diverse biological roles, including antifungal and antibacterial (Fukushima *et* *al*., 2011). Two other singletons annotated “ent-kaurene oxidase” belong to the P450 class and were conserved. Interestingly, a comparison with the Arabidopsis proteome database revealed homologies with ENT-KO, a member of the CYP701A subfamily involved in the gibberellin biosynthetic pathway (Sawada *et* *al*., 2008; Achard & Genschik, 2009; X.-H. Gao *et* *al*., 2011). Moreover, another gene (corresponding to CG-R02f_045_J11, Mtr.31291.1.S1_at, OS07G39270) annotated as GeranylGeranyl Pyrophosphate Synthase, is homologous to *AtGGPS1*, which is also involved in Gibberellin biosynthesis (Okada *et* *al*., 2000). These results are consistent with the Gibberellin biosynthesis regulation occurring in AM (Güimil *et* *al*., 2005; Gomez *et* *al*., 2009; Schäfer *et* *al*., 2009; Fiorilli *et* *al*., 2009; Hogekamp *et* *al*., 2011) and with the postulated role of this phytohormone as a compatibility factor in AM [27].

Another important group that was conserved were transporters: eight *C. glauca* genes belong to this category. Among them is the aforementioned MtPT4 (Mtr.43062.1.S1_at; [28] and its orthologues in rice (OsPT11; OS04G10800; [29]) and *C. glauca* (CgPT4, CG-GI1f_006_M09)) encoding a high affinity phosphate transporter specifically expressed in the peri-arbuscular membrane and responsible for the symbiotic transport of phosphate in *M. truncatula* [30]. Another transporter (CGCL918Contig1) shared 73% identity with MtNIP1, an aquaporin specifically expressed in cells containing arbuscules [31], which has been suggested as being involved in inorganic N uptake into plant cytoplasm [31], [32]. Other genes related to transport encode putative oligopeptide transporters potentially involved in the intake of small peptides produced by the degradation of fungal proteins during the senescence of arbuscules, or in the intake of signal peptides [18]. Two putative members of the ABC-transporter family are also among the conserved genes. CG-N02f_013_P06 shares 80% homology with MtSTR2 (for stunted arbuscules; Zhang *et* *al*., 2010). MtSTR2 interacts with MtSTR to form a functional heterodimeric transporter that co-localises at the peri-arbuscular membrane and is essential for arbuscule development [33]. CGCL1417Contig1 presents 62% identity with AtPGP1 (P-GlycoProtein 1), 63% with AtPGP4 and 63% with AtPGP16. These members of the P-GLYCOPROTEIN (PGP) transporters family are able to transport a wide range of molecules [34].

Another conserved gene cluster corresponds to chitinases (4 *C. glauca* unigenes). Interestingly, CGCL506Contig1 presents 84% similarity with MtCHITIII-3, which is specifically expressed in cells containing arbuscules in *M. truncatula* [35]. Disruption of its expression resulted in a higher root colonisation by *G. intraradices* [36]. This chitinase may be involved in the modulation of chitin elicitors, and have an impact on signalling between the plant and fungus.

Genes involved in lipid metabolism are also conserved between the three species; this is in accordance with the important role played by lipid metabolism during synthesis of the peri-arbuscular membrane concomitant to internalisation of the fungi, as well as in recycling lipids from degenerating arbuscules [2]. A purple acid phosphatase, CG-GI1f_003_A02, sharing 86% identity with AtPAP10 and 88% identity with MtPAP1, was also identified. These proteins are involved in phosphate nutrition probably through phytate degradation [37], [38]. Within conserved elements, we identified several signalling components such as protein kinases, a U-box protein from the same family as LIN, a protein involved in nodulation [39], and transcription factors from the GRAS and AP2/ERF family.

In conclusion, our study highlights key biological processes that were conserved throughout plant evolution, and that are probably essential for AM establishment and functioning.

### Comparison of gene expression in AM, rhizobial and actinorhizal symbioses

**Figure 3:**
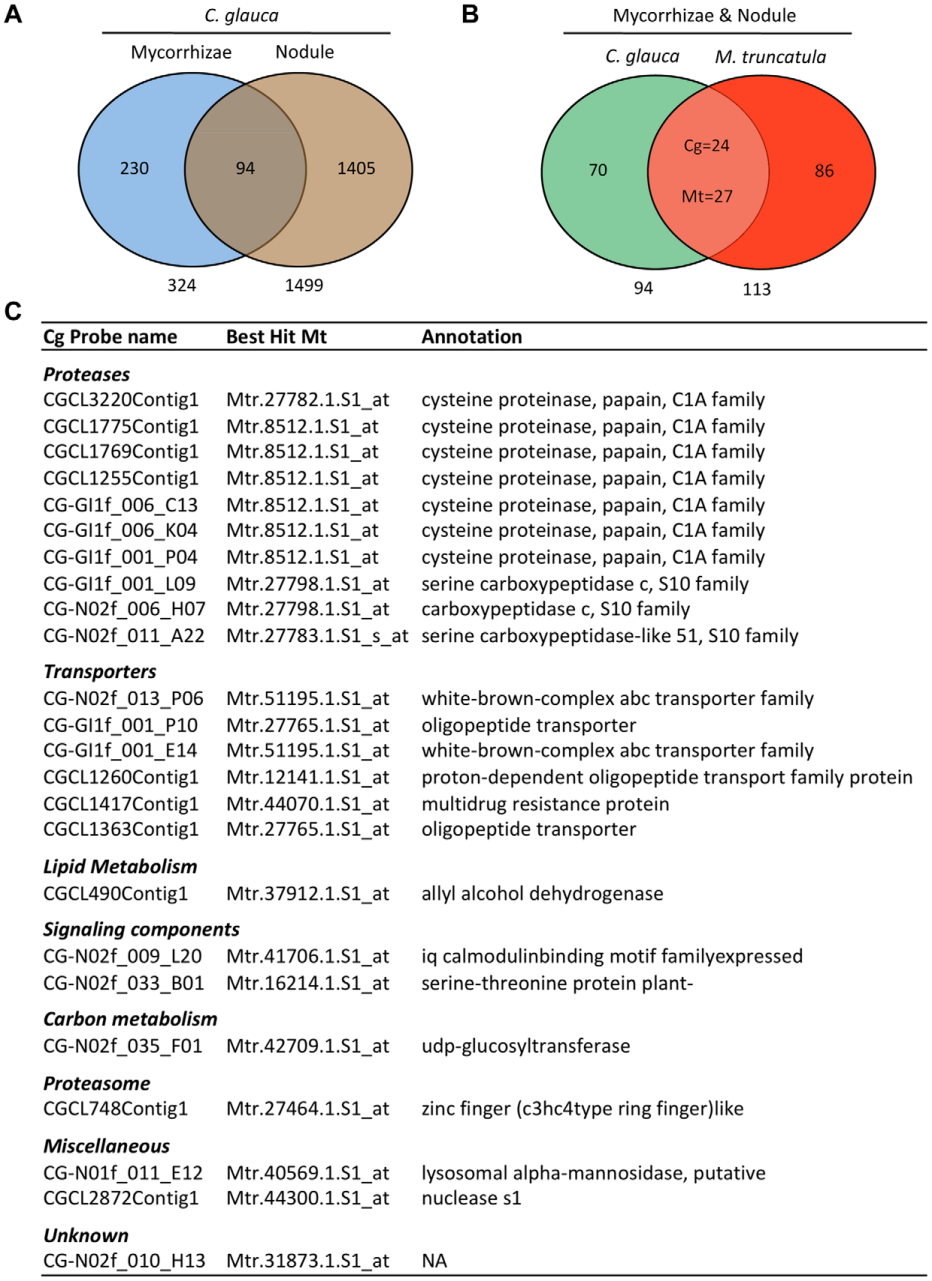
Conservation of gene expression in AM and root-nodule symbioses. (A) Transcriptomic comparison between *C. glauca* genes up-regulated in AM and actinorhizal nodules; (B) Conservation of genes up-regulated in both AM and nodules in *C. glauca* and *M. truncatula*; (C) Functional classification of the 24 conserved genes up-regulated during AM, actinorhizal and legume-rhizobium symbioses.

In order to analyse the potential conservation of the molecular mechanisms involved in AM and actinorhizal symbioses, we compared genes induced in these two symbioses in *C. glauca*. A simple spreadsheet application named Casuarina Transcriptome Compendium (CTC; Table S5) was created for comparative transcriptomics in *C. glauca* (for guidelines, see File S1). CTC allowed us to identify 94 genes up-regulated both in AM and actinorhizal nodules (FC> = 2, p-value< = 0.01, Figure 3A; Table S6). Functional classes recovered were partially similar to those found when comparing AM and rhizobal symbioses in Legumes [19], [40]. RT-qPCR was used to confirm the induction of a subset of these genes in both interactions (Table S6).

In order to compare the set of genes involved in AM and root nodule symbioses in both legumes and actinorhizal plants, we compared the genes up-regulated in AM and actinorhizal symbioses in *C. glauca* to those up-regulated in both nodules and AM recently identified in the model legume *M. truncatula* (respectively 51 K and 61 K Affymetrix geneChip) [14], [15]. Twenty-four *C. glauca* genes induced in AM roots and nodules (MycUp/NodUp) presented significant sequence homology with *M. truncatula* MycUp/NodUp genes (Figure 3B–C; Table S7). These genes might represent part of the heart of endosymbioses, conserved together in the ancestral AM symbiosis, legume-rhizobial and actinorhizal symbioses.

Once again, genes encoding proteases formed the largest cluster (10/24), suggesting that proteases play a significant common role in the three endosymbioses. Interestingly, mutant screenings performed on model legumes did not yield any gene encoding protease involved in rhizobial or AM symbioses, either because mutants in these genes are lethal or because a high redundancy level is present. Maintenance of functional redundancy may reflect a need for very high expression levels for these genes in the context of endosymbioses [41]. Gene encoding transporters represented the second largest group. This group included the *C. glauca STR2* homologue represented by 2 probes (CG-N02f_013_P06 and CG-GI1f_001_E14) corresponding to the same unigen. Zhang *et* *al*. (2010) did not report any phenotype during nodulation in the *mtstr2* mutant. Our finding that this gene was among the core endosymbiotic gene set suggests that it may still play a subtler role in nodulation. Genes encoding peptide transporters and PGP family transporters were also up-regulated during all 3 endosymbioses.

In conclusion, our work revealed genes that are induced in all three major plant endosymbioses: the ancient AM symbiosis, and the more recent RNS. This list represents genes probably linked to processes such as nutrient exchange, infection, and intracellular accommodation of the microsymbiont, and reflects the molecular tinkering that took place during evolution of nodulation using parts of ancestral AM mechanisms. Recycling signal transduction elements from AM to form RNS has previously been reported [7], [12], [17]. The corresponding genes were not recovered in our work as they are often not transcriptionally regulated (Table S8). The genes we identified were strongly up-regulated in all endosymbioses and probably correspond to the end targets of the endosymbiotic programme. Further functional characterisation of these genes is needed to understand their precise role in the three different endosymbioses and to explain how they were recruited during the evolution of RNS.

## Materials and Methods

### Plant and fungal material

Initial cultures of *Daucus Carota* and *Glomus intraradices* DAOM 197198 were provided by G. Bécard (Laboratory of Cell Surfaces and Plant Signalling, UMR CNRS-Paul Sabatier University, Toulouse, France). *C. glauca* seeds were purchased from CSIRO (Australia). Seeds were germinated in sterile conditions and grown for three weeks in hydroponics containing a modified BD medium [42]. Plants were then transferred to pots containing a sterile sand: soil mixture, inoculated with *G. intraradices* as described [12] and grown in a culture chamber at 25°C, with an 18/6-h photoperiod.

### Root colonisation analysis

AM structures were observed on roots stained with Trypan blue or Uvitex2B as described [43]. Mycorrhization rates were evaluated every 3 days starting at 15 dpi on root systems stained with Trypan blue and observed using a DMRB microscope (Leica). Mycorrhization was scored on 5 root systems using the gridline intersect method [44] and at least 100 intersections were scored per sample. Mycorrhizal structures were analysed on root samples stained with Uvitex2B using a 510 META confocal microscope (Zeiss) and a Plan Apochromat ×63/1.4 oil or a Plan Neofluar x25/0.8 oil objective. Two independent acquisitions were performed, one at 760/435–485 (bi-photon excitation/emission) for Uvitex2B and one at 488/533–619 for autofluorescence.

### Gene expression analyses

Roots were harvested 45 days after inoculation by *G. intraradices*. Control (uninoculated) plants were grown for the same time in the same medium. Three biological replicates were performed for each condition. The presence of AM structures was analysed on control and inoculated roots before RNA extraction. RNA extraction, cDNA synthesis and hybridisation on *C.glauca* microarray were conducted as described [43]. Data were scanned, normalised (array normalisation: median of each array, probe normalisation: median of the samples for each probe-set) and absolute values and flags were extracted independently for the two experiments. A mean was calculated by averaging triplicate absolute expression values for each condition. For each respective experiment, pair-wise comparison fold changes (FC) were calculated successively by applying a ratio between the conditions, applying a log2 transformation, and calculating the opposite inverse for the values smaller than 1. For each experiment, a p-value was calculated based on a t-test assuming that the variances were equal, using the MeV software package (http://www.tm4.org/mev/). A gene was considered to be differentially expressed in each independent experiment if it could satisfy the following conditions: at least two “Present” calls in at least one of the condition triplicates, a fold change greater than or equal to 2, and a p-value less than or equal to 0.01. Q-PCR experiments were performed as described [17] using primers listed in Table S9.

### Sequence analyses

EST sequences were retrieved respectively from a previously described database [17] for *C. glauca;* http://bioinfo.noble.org/mt-affyprobeset-mapping/Medicago_Affy_Consensus_Seqs.fasta for *M. truncatula* and http://bioinfo.noble.org/mt-affyprobeset-mapping/Medicago_Affy_Consensus_Seqs.fasta *: O. sativa*:

http://bioinformatics.psb.ugent.be/plaza/download for *O. sativa*. Sequences from *C. glauca* were re-annotated using Blast2Go [45]. Tblastx2 was used to check trans-species sequence homologies between genes with an e-value cut-off of 1e^−10^.

## Supporting Information

File S1 **CTC tutorial.**
(PPT)

Table S1 **Validation of** ***C. glauca*** **microarray gene expression data using real time PCR.**
(XLS)

Table S2 ***C. glauca*** **genes induced in response to AM symbiosis.** Genes are classified by predicted function according to their annotation and sorted by descending induction level in AM.
(XLS)

Table S3 ***G. intraradices*** **genes expressed in** ***C. glauca*** **AM roots.** Genes were identified by blast against the *G. intraradices* EST database, with the condition of obtaining an e-value greater than that obtained against the plant proteome.
(XLS)

Table S4 **AM-induced genes sharing significant sequence conservation in** ***C. glauca*** **,** ***M. truncatula*** **and** ***O. sativa.*** Data on *M. truncatula* come from additional File 1 of Gomez *et* *al*., 2009. Data on *O. sativa* come from Table S1 of Güimil *et* *al*. (2005). Sheet 1 (Cg vs Mt vs Os) contains genes classified by predicted function according to their annotation and sorted by descending induction level in AM. Sheet 2 (Cg versus Mt Blast Result) and 3 (Cg versus Os Blast Result) contain detailed blast results corresponding to comparison between *C.glauca* and *M. truncatula,* and between *C.glauca* and *O. sativa* respectively.
(XLS)

Table S5 **CTC, Casuarina Transcriptome Compendium.**
(XLSX)

Table S6 **Genes induced during both AM and actinorhizal symbiosis in** ***C. glauca*** **.** Gene expression data on actinorhizal symbiosis correspond to nodules 21 days post inoculation compared to control in *C. glauca* (Hocher *et* *al.,* 2011) and validation of the microarray data using real time PCR for a subset of genes.
(XLS)

Table S7 **Conserved genes up-regulated during AM, actinorhizal and rhizobial symbioses.** Gene expression data on rhizobial symbiosis correspond to nodules 14 days post inoculation compared to control in *M. truncatula* (Benedito *et* *al*., 2008). Sheet 1 (CgMYCupNODup vs MtMYCupNODup) contains genes classified by predicted function according to their annotation and sorted by descending induction level in AM. Sheet 2 (Blast Results) contains blast results.
(XLS)

Table S8 **Expression in** ***C. glauca*** **actinorhizal nodules and AM roots of genes sharing sequence identity with genes involved in Nod factor signal transduction.**
(XLS)

Table S9 **Sequences of Primers used for Real Time PCR.**
(XLS)
