# Supplementary material for: Heart of Endosymbioses: Transcriptomics Reveals a Conserved Genetic Program among Arbuscular Mycorrhizal, Actinorhizal and Legume-Rhizobial Symbioses
Source: PLoS One. 2012 Sep 6;7(9):e44742. doi: 10.1371/journal.pone.0044742 (PMC3435296; doi:10.1371/journal.pone.0044742)
Supplement: File S1 — CTC tutorial. (PPT) [file pone.0044742.s001.ppt]

## Slide 1
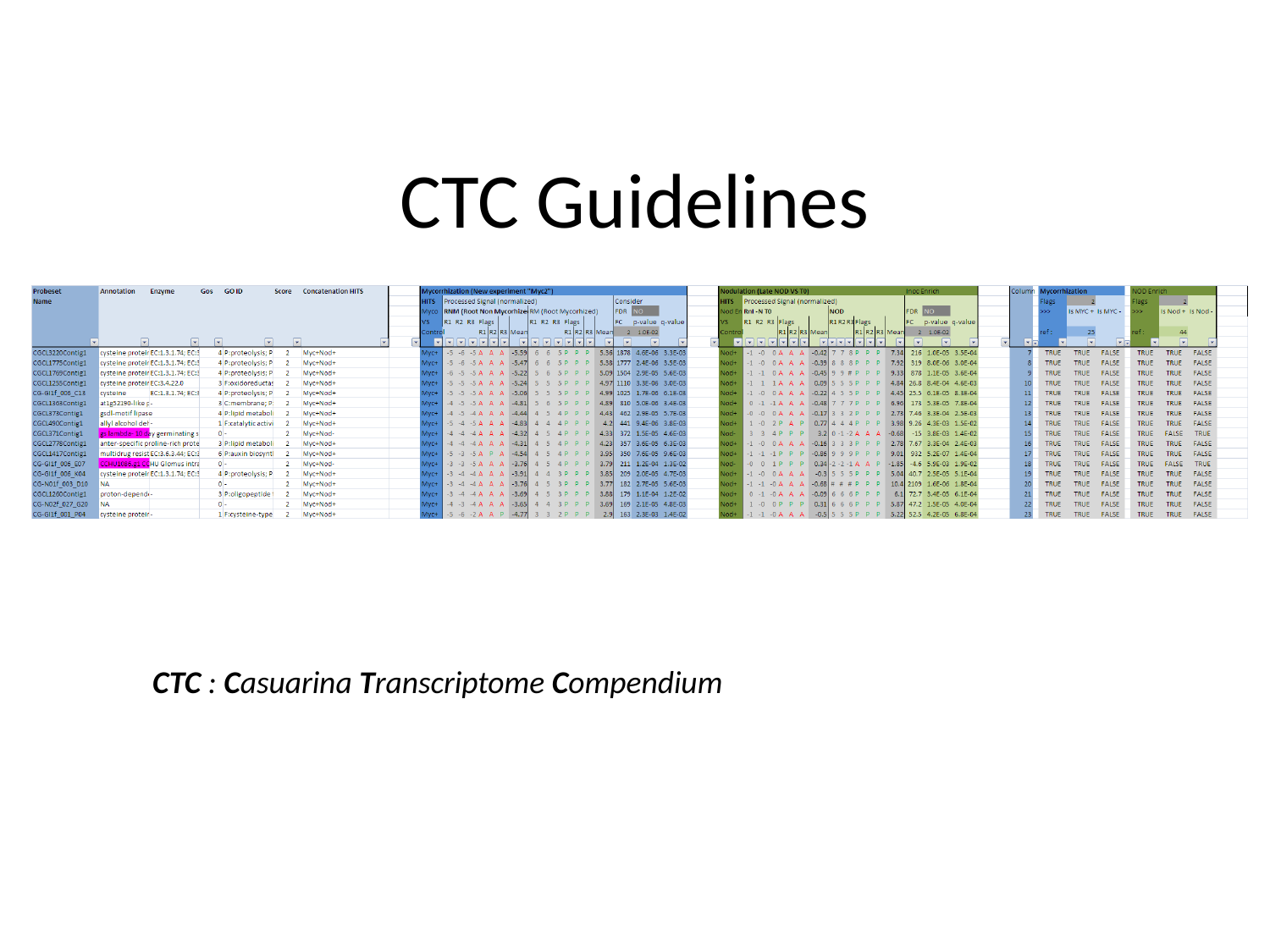

# CTC Guidelines
CTC : Casuarina Transcriptome Compendium

## Slide 2
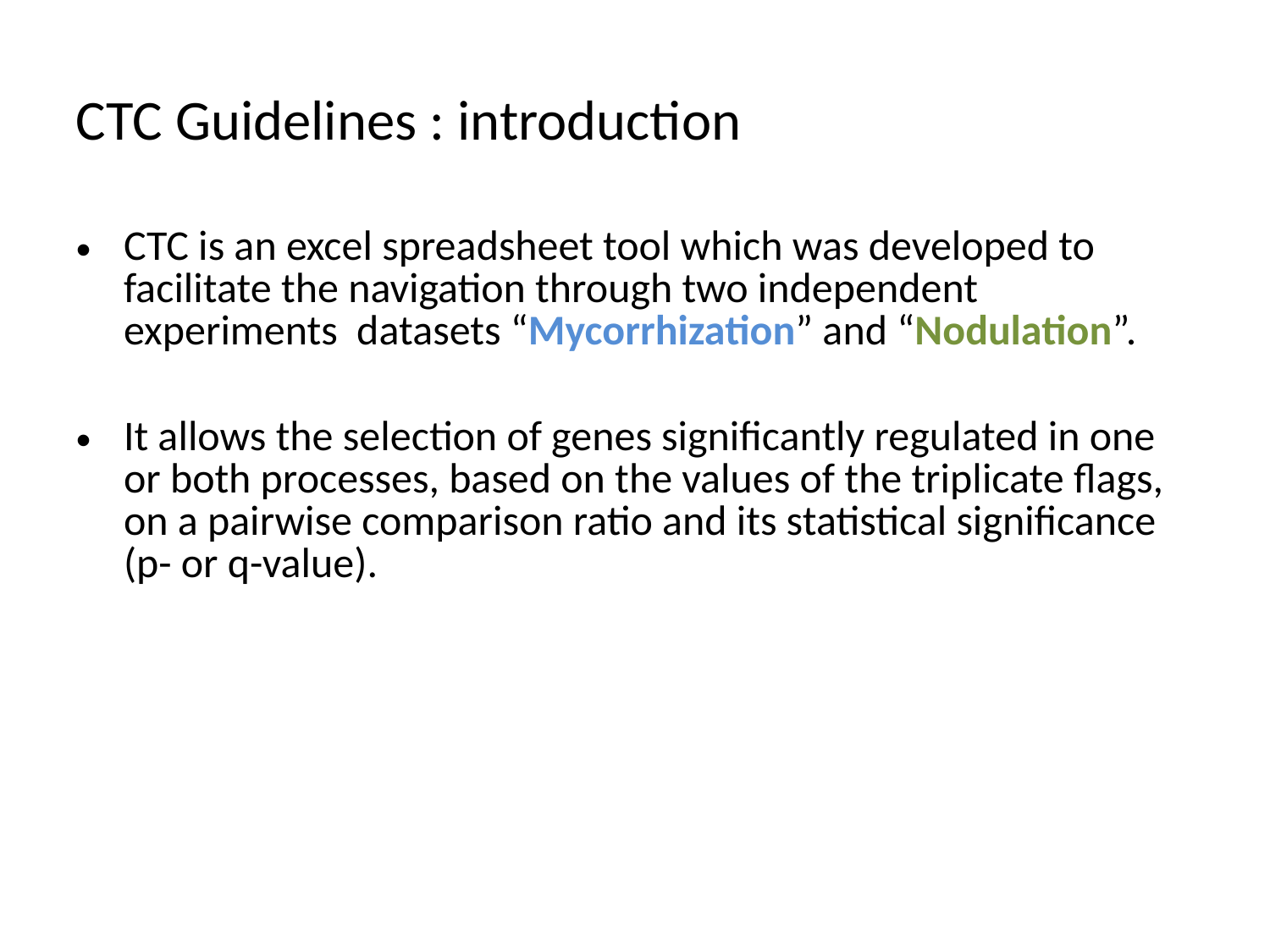

# CTC Guidelines : introduction
CTC is an excel spreadsheet tool which was developed to facilitate the navigation through two independent experiments datasets “Mycorrhization” and “Nodulation”.
It allows the selection of genes significantly regulated in one or both processes, based on the values of the triplicate flags, on a pairwise comparison ratio and its statistical significance (p- or q-value).

## Slide 3
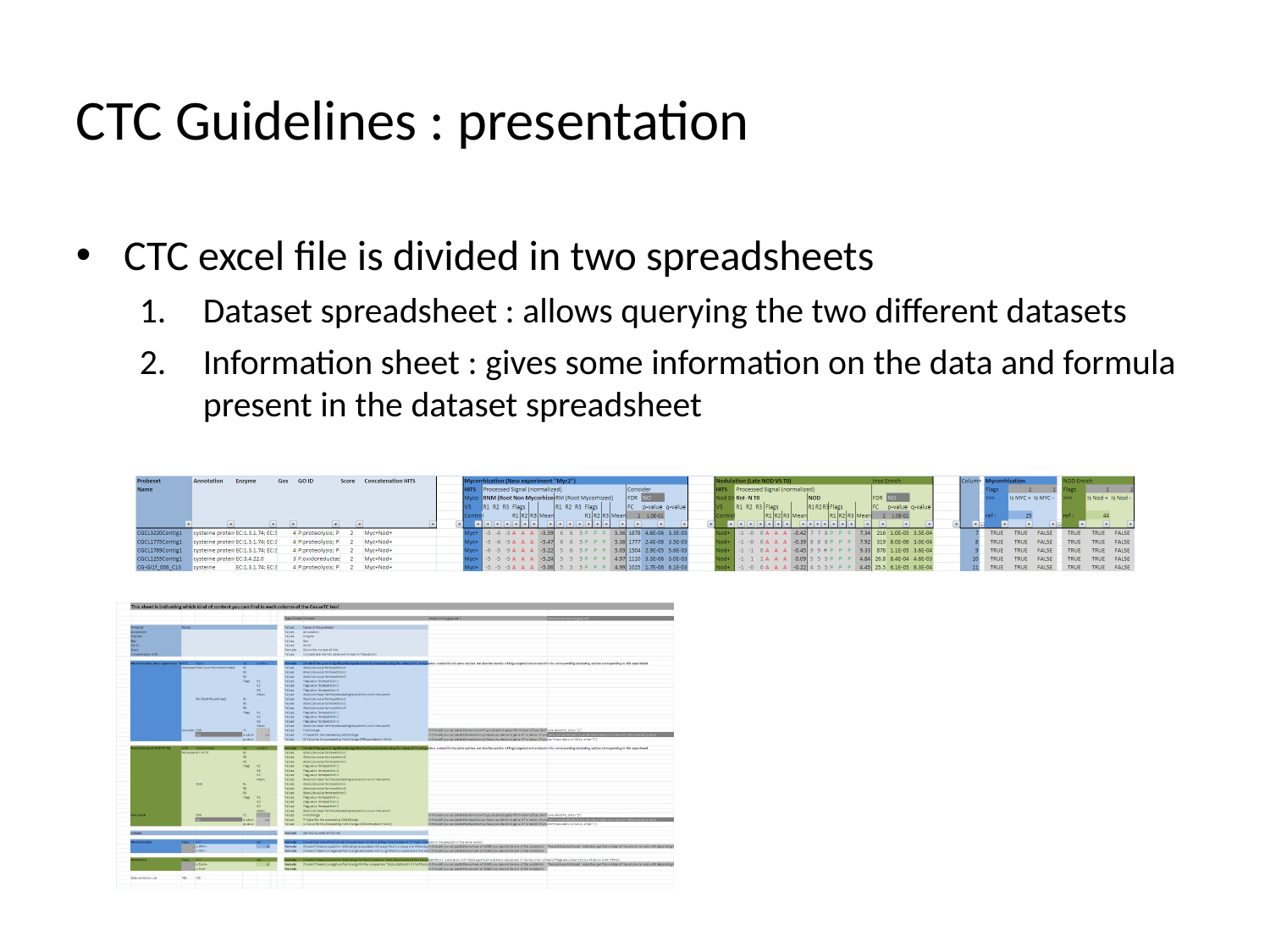

# CTC Guidelines : presentation
CTC excel file is divided in two spreadsheets
Dataset spreadsheet : allows querying the two different datasets
Information sheet : gives some information on the data and formula present in the dataset spreadsheet

## Slide 4
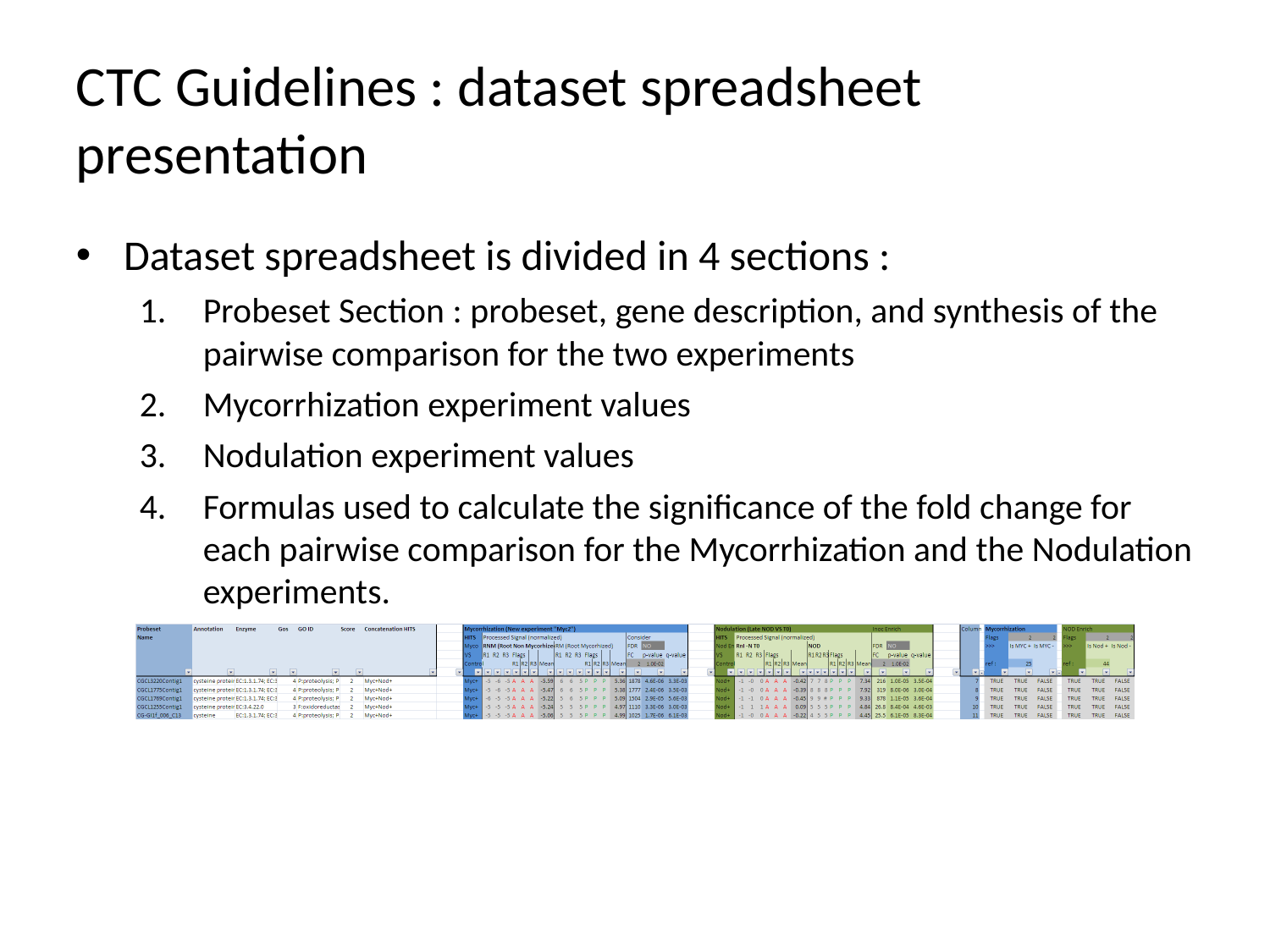

# CTC Guidelines : dataset spreadsheet presentation
Dataset spreadsheet is divided in 4 sections :
Probeset Section : probeset, gene description, and synthesis of the pairwise comparison for the two experiments
Mycorrhization experiment values
Nodulation experiment values
Formulas used to calculate the significance of the fold change for each pairwise comparison for the Mycorrhization and the Nodulation experiments.

## Slide 5
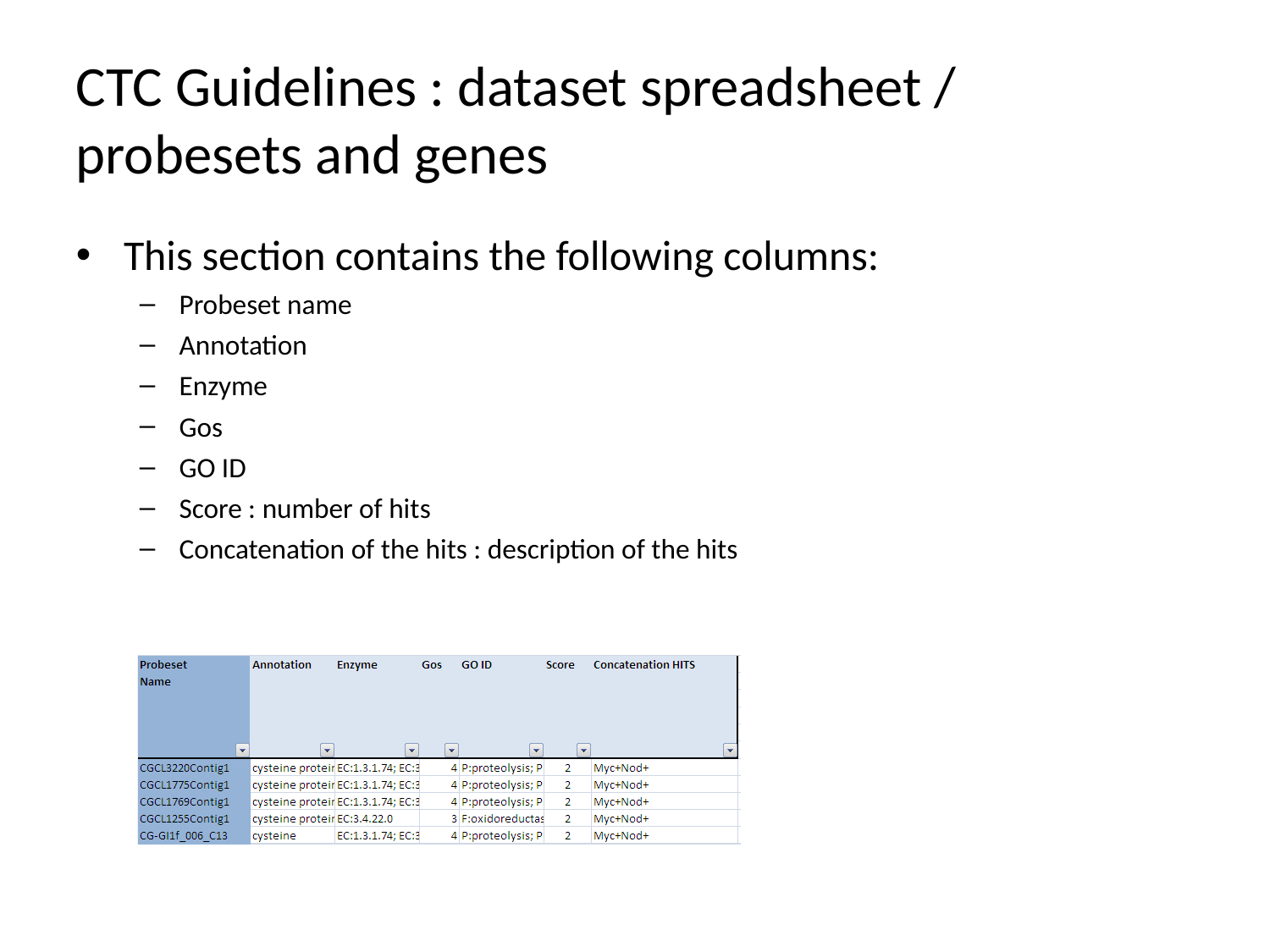

# CTC Guidelines : dataset spreadsheet / probesets and genes
This section contains the following columns:
Probeset name
Annotation
Enzyme
Gos
GO ID
Score : number of hits
Concatenation of the hits : description of the hits

## Slide 6
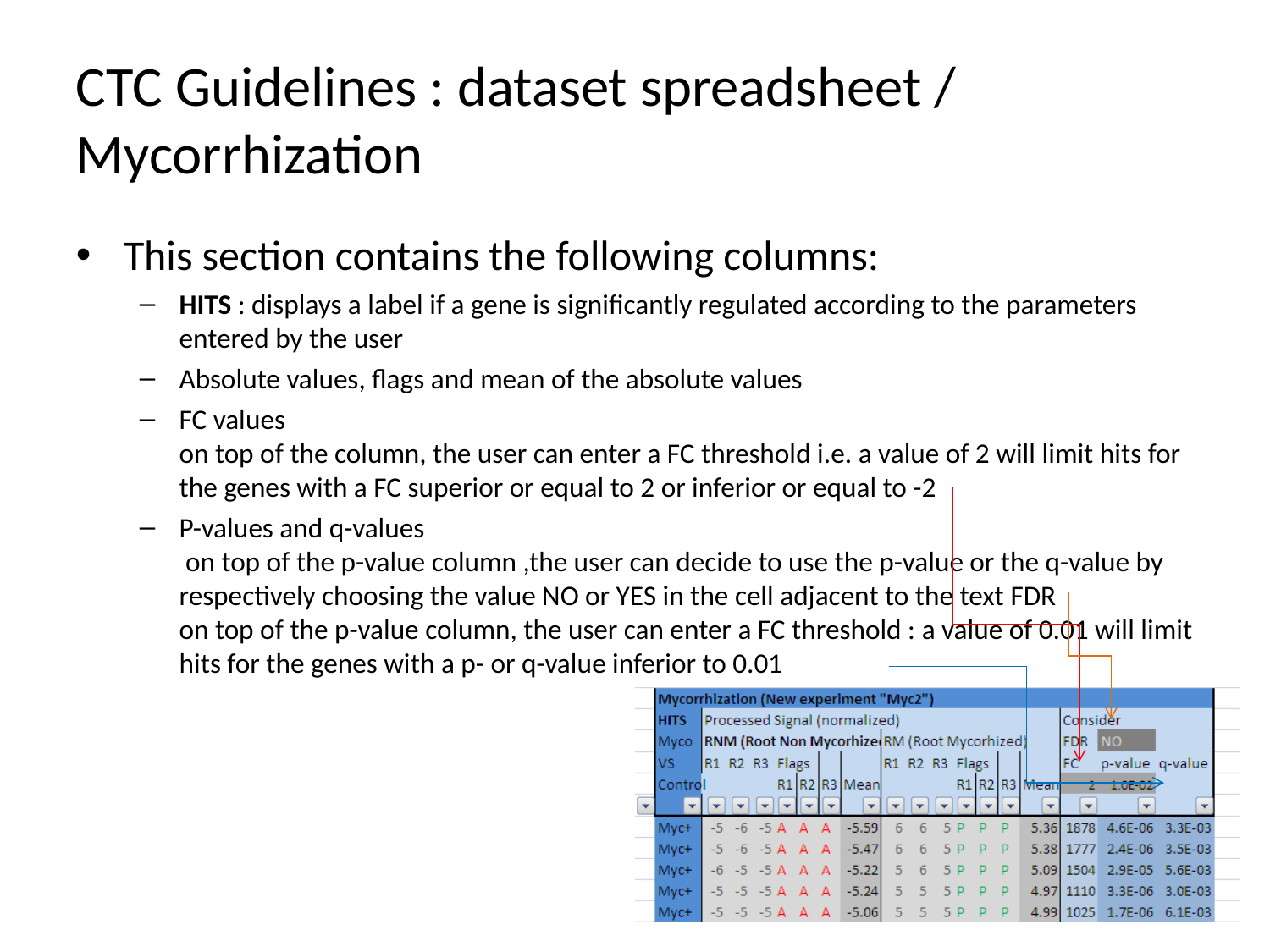

# CTC Guidelines : dataset spreadsheet / Mycorrhization
This section contains the following columns:
HITS : displays a label if a gene is significantly regulated according to the parameters entered by the user
Absolute values, flags and mean of the absolute values
FC values
on top of the column, the user can enter a FC threshold i.e. a value of 2 will limit hits for the genes with a FC superior or equal to 2 or inferior or equal to -2
P-values and q-values
 on top of the p-value column ,the user can decide to use the p-value or the q-value by respectively choosing the value NO or YES in the cell adjacent to the text FDR
on top of the p-value column, the user can enter a FC threshold : a value of 0.01 will limit hits for the genes with a p- or q-value inferior to 0.01

## Slide 7
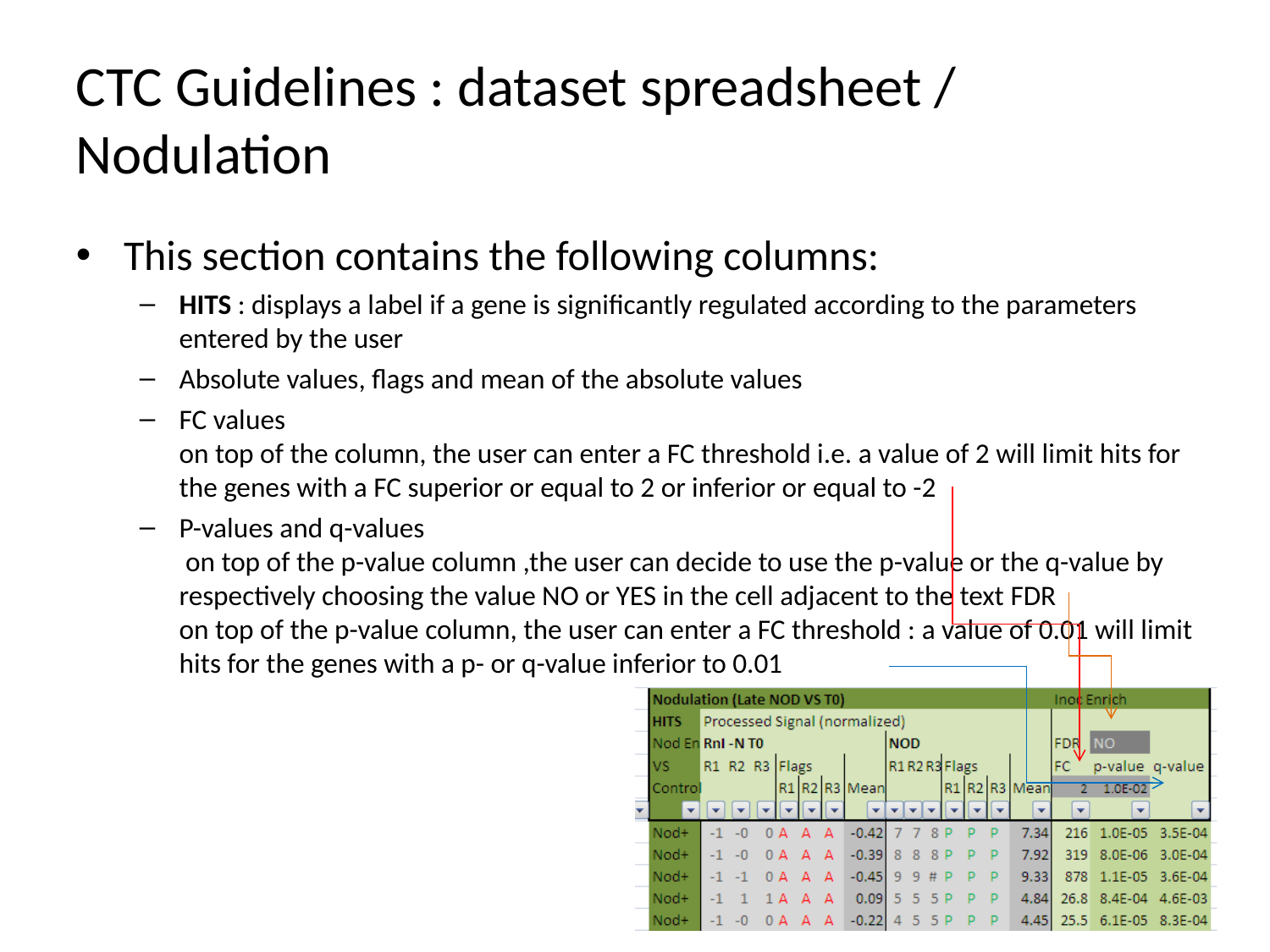

# CTC Guidelines : dataset spreadsheet / Nodulation
This section contains the following columns:
HITS : displays a label if a gene is significantly regulated according to the parameters entered by the user
Absolute values, flags and mean of the absolute values
FC values
on top of the column, the user can enter a FC threshold i.e. a value of 2 will limit hits for the genes with a FC superior or equal to 2 or inferior or equal to -2
P-values and q-values
 on top of the p-value column ,the user can decide to use the p-value or the q-value by respectively choosing the value NO or YES in the cell adjacent to the text FDR
on top of the p-value column, the user can enter a FC threshold : a value of 0.01 will limit hits for the genes with a p- or q-value inferior to 0.01

## Slide 8
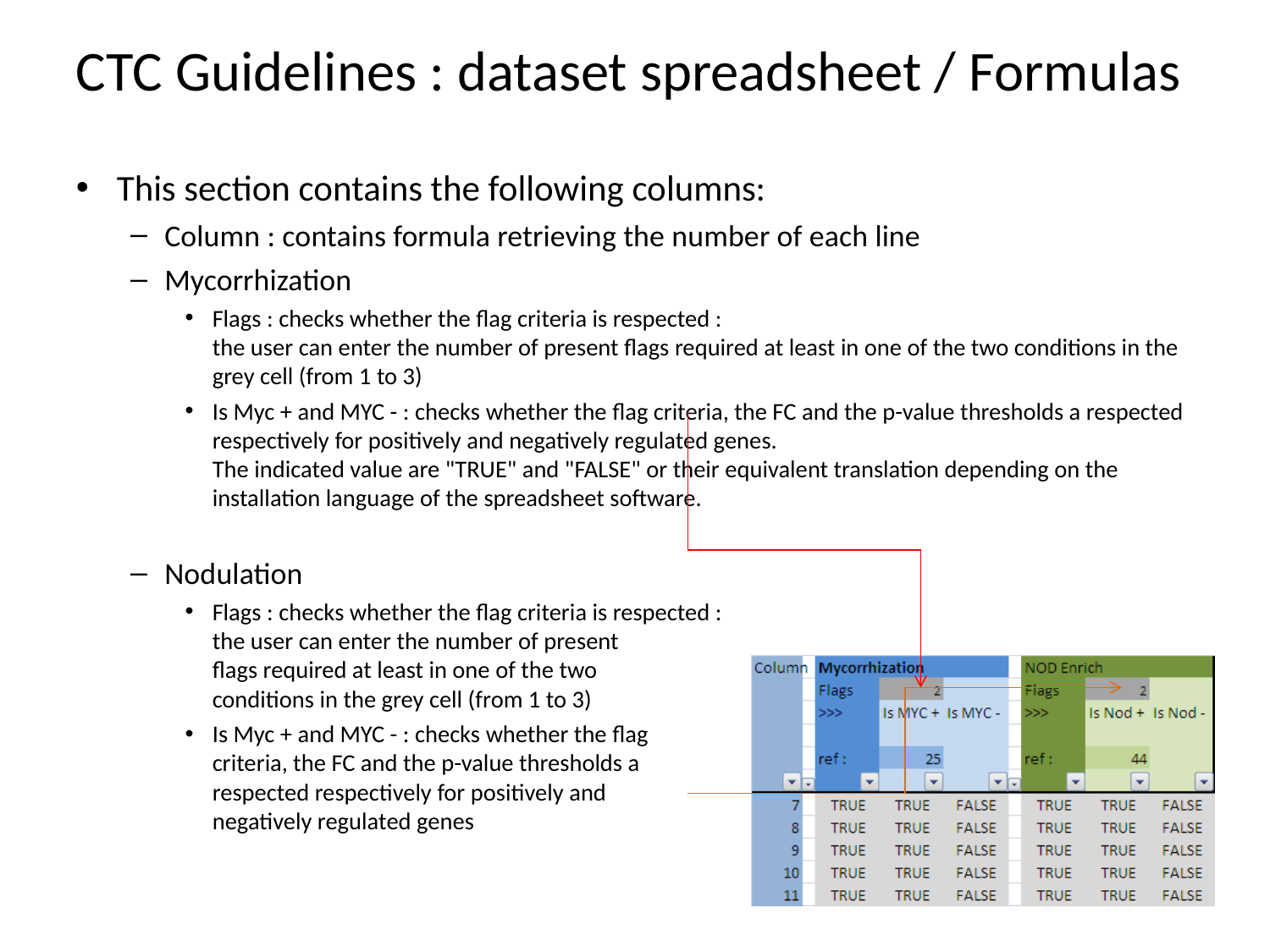

# CTC Guidelines : dataset spreadsheet / Formulas
This section contains the following columns:
Column : contains formula retrieving the number of each line
Mycorrhization
Flags : checks whether the flag criteria is respected :
the user can enter the number of present flags required at least in one of the two conditions in the grey cell (from 1 to 3)
Is Myc + and MYC - : checks whether the flag criteria, the FC and the p-value thresholds a respected respectively for positively and negatively regulated genes.
The indicated value are "TRUE" and "FALSE" or their equivalent translation depending on the installation language of the spreadsheet software.
Nodulation
Flags : checks whether the flag criteria is respected :
the user can enter the number of present
flags required at least in one of the two
conditions in the grey cell (from 1 to 3)
Is Myc + and MYC - : checks whether the flag
criteria, the FC and the p-value thresholds a
respected respectively for positively and
negatively regulated genes
